# Value of Care: An Exploratory Qualitative Study with Doctors and Patients

**DOI:** 10.3390/ejihpe13070084

**Published:** 2023-06-21

**Authors:** Leda Marino, Vincenza Capone

**Affiliations:** Department of Humanities, University of Naples Federico II, 80100 Naples, Italy; vincenza.capone@unina.it

**Keywords:** value, physicians, patients, healthcare organisations, psychosocial perspective, qualitative study, doctor–patient relationship

## Abstract

The concept of value in healthcare is mainly based on economic and financial aspects. However, the literature has emphasised the need to investigate value from other perspectives. The present study aimed to explore the views of physicians and patients on the value of healthcare, and to examine in depth the psychosocial and organisational elements that have emerged but that need to be investigated more. Therefore, two qualitative studies were performed, in which 69 physicians and 111 patients participated. The data were analysed using content analysis and text mining using t-lab software. The results revealed common elements between the two healthcare actors that constitute value in healthcare, including competence, professionalism, and soft skills like communication and empathy. Furthermore, the importance of functioning health services and effective organisational culture in local healthcare and investment emerged. These findings can guide healthcare organisations to consider the potential psychosocial factors related to value in healthcare, which affect organisation in terms of costs and healthcare relationships. In addition, these findings are a first step in filling the gap found in the literature regarding the consideration of value from a non-economic perspective and the difficulty of defining and measuring it.

## 1. Introduction

From an economic and marketing perspective, value (historically considered a financial resource) has recently been considered as a relational aspect in its shared matrix [1].

As Lewis [2] affirmed, “It takes two to tango”; shared health goals are needed for truly person-centred care (p. 212), tailoring treatment to the individual’s health goals and context to promote self-management care (p. 213), in line with the health engagement perspective [3].

The existing literature, e.g., [4,5], mentions but does not address the psychosocial perspective (which is our theoretical framework, in connection with organisational psychology), despite highlighting the need to explore the topic of value in healthcare from different approaches beyond economics.

For example, Porter ref. [1] (p. 325) stated that “most companies are stuck in a mindset where social issues are at the periphery, not the center”, including healthcare organisations. As long as business and society are divided, the principle of shared value whereby “the creation of economic value also creates value for society by addressing its needs and challenges” [1] (p. 325) will not occur. According to the authors, stakeholder needs should define the line of markets as much as conventional economic needs. Therefore, value, to be valued, must be shared. According to the authors, shared value is a virtuous circle; increasing value in one area creates opportunities in others [1] (p. 329). However, as Vargo and Lush [6] underline, what “value” means between different theoretical perspectives remains problematic and unexplored, and needs investigation. Furthermore, the literature [7,8] points out that it is unclear what “healthcare customers” think and feel when they collaborate with “healthcare providers” to create value. Moreover, the absence of studies on patients’ and physicians’ values from psychosocial perspectives leaves quite a few gaps in the scientific debate [7]. Several studies [9,10,11,12,13] performed from a financial marketing perspective cited and acknowledged psychological aspects such as partnership, personal resources, collaboration between “health clients” and “providers”, and communication, and considered that value could also be created in the encounter between physicians and patients in order to achieve better health outcomes (such as psychophysical wellbeing or better compliance) and reduce human and financial costs within the available health service. Beyond that, they did not explore this and other aspects in a psychosocial framework. This does not allow scholars to have a solid ground when they inevitably wrestle with psychosocial issues, e.g., health communication and relational aspects that occur and characterise the care encounter [14,15].

Comprehension of value in a non-economic framework is necessary since it impacts the functioning of the healthcare organisation [16]. Therefore, from a psychosocial perspective [17], what is value in healthcare? To try to give an answer, two exploratory qualitative studies were implemented related to the views of physicians and patients on value in healthcare.

### Aim of the Study

This qualitative study aims to investigate the concept of value in healthcare from a psychosocial perspective, from the physicians’ and patients’ points of view as the actors in healthcare organisations. The study is a part of a doctoral research project from December 2019 to November 2022. The general project aims to develop a measurement addressed to physicians and patients to detect psychosocial aspects from the Value-Based Healthcare framework [18].

## 2. Materials and Methods

### 2.1. Procedures

Two self-report studies were conducted, and questions were implemented using Google^®^’s Platform. The first related to physicians (from January to April 2021), and the second to patients (from May to September 2021). Participants were recruited through a convenience sampling strategy and, beyond being above the age of 18, the patients’ inclusion criterion was having had a medical consultation during the last month. Participation was anonymous, no incentive was given, and informed consent was obtained from all participants through a specific section in the questionnaire. All procedures followed were in accordance with ethical standards and the Helsinki Declaration (2013) [19], and the protocol was approved by the *Ethics Committee of Psychological Research of the Department of Humanities of the University of Naples Federico II* (prot. 18/2022) (The Ethical Committee of Psychological Research of the Department of Humanities of the University of Naples Federico II met to analyse the request of Dr. Leda Marino, regarding her request for attesting conformity to ethical norms in the research project entitled “Health perceived value in the Value-Based Healthcare (VBHC) perspective”. The project was examined by the members of the Ethics Committee for Psychological Research according to the procedures agreed upon by the Department, and its compliance with ethical standards was certified). All participants were given the option to withdraw at any moment.

Participants were asked one open-ended question following some general formulae, e.g., “write the first things that come to your mind while thinking about value in healthcare…” [20] (p. 225). Participants were asked to write down what expresses meaning in relation to the proposed concept, from their point of view. In addition, free word association tasks are easy to understand and can be performed quickly, which allows other procedures to be added to the questionnaires to specify data collection [21].

At the same time, the easy nature of the task allowed the implementation of the survey via an online platform, to respect the Italian restrictions related to COVID-19 management. During the pre-pandemic era, the aim was to administer a semi-structured interview to physicians and patients within healthcare, to explore the most relevant psychosocial aspects of value in their discursive responses.

During the coronavirus outbreak in Italy, it was impossible to access any healthcare services. Hence, the problem was remedied with a *free written word association technique* (self-administered) [20,21]. Thus, we asked participants the following question:

“*What does value in healthcare currently consist in your opinion*? Write down the first things you consider” (translate from Italian).

Two different pieces of software were used for the data analysis. Socio-demographic information data analysis was performed using IBM SPSS Statistics^®^ 26.0, while T-Lab 21 software was used for the qualitative data analysis. We started from the lexis’ disambiguation [22].

**Figure 1:**
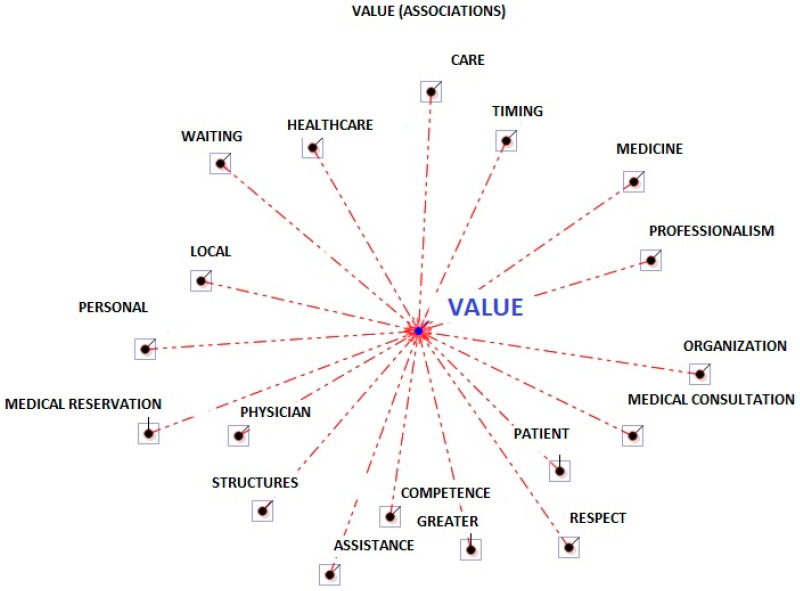
Radial diagram of the lemmas’ associations for physicians and patients. The selected lemma (value) is placed in the centre, and the others are distributed around it proportional to their degree of association (T-Lab 21).

**Figure 2:**
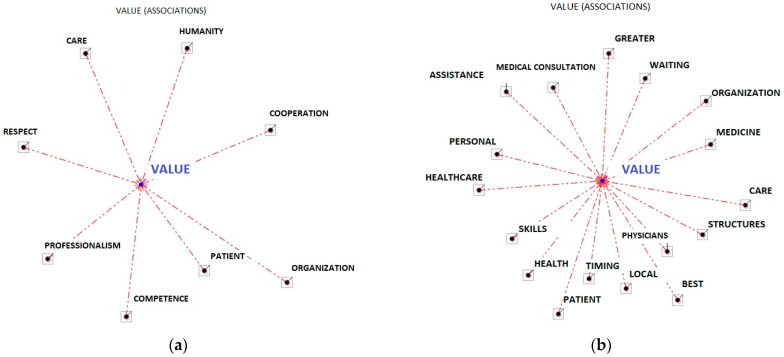
(**a**) Radial diagram of the lemmas’ associations for physicians; (**b**) radial diagram of the lemmas’ associations for patients.

There were some cases of homographs, e.g., the word “organisation” as “healthcare” or “structure”. Lexicalisation was performed, and strings were imported as a new *txt* corpus from the analysis of polyurematic locutions (e.g., ‘public health’). The corpus was small, and so only lexical units with an average frequency rank [23], i.e., 4, were chosen. Considering this keyword selection criterion, there turned out to be 81. The total analysis corpus consisted of 15,765 occurrences and 1439 lemmas (Lemmatisation processes regard the reduction of corpus words to their respective headwords (i.e., lemmas). In the linguistic dictionaries that we may consult, every entry corresponds to a lemma that, generally, defines a set of words with the same lexical root (or lexeme) and that belongs to the same grammatical category (verbs, adjectives, etc.). As a rule, lemmatisation entails that verb forms are taken back to the base form, nouns to the singular form, and so on [22]). Then, the word association test and the association indexes were carried out. The association and output analyses were evaluated by 3 independent health and organisational psychology expert judges. Specifically, an analysis of occurrence and co-occurrence was carried out (see Table 1) to evaluate the frequency with which a word appeared in a participant’s sentence, and whether two or more words emerged in the same context (e.g., homographs cases). Subsequently, through the word association analysis, the software proceeded to construct a map (the radial map, see Figure 1 and Figure 2), evaluating two aspects: a list of lexical units, and the relationships between the units. This process created a word association index shown on the radial map, with the keyword (*Value*) in the centre, and the other related words distributed around each other with a distance proportional to their association with the keyword (association index; see Table 2). The more words that were near the keyword, the more frequent their co-occurrence. The strength of the associations between keywords was assessed using the χ^2^ test (see Table 2) [23].

### 2.2. Participants in Physicians’ Study

A total of 101 physicians were invited, and 69 of them participated in the research (all from the Campania region, southern Italy). In addition to age of majority, the inclusion criteria included being or having been a physician in a public hospital and having completed a medical specialisation. Physicians in training and employees of private health services were excluded, because the literature defines these contexts as culturally different from that of the public health companies that are the subject of our work [13].

The information was checked by means of two initial questions relating to these issues. This was because the question “state your medical specialization” could not be made mandatory for ethical reasons. In fact, a response rate of 25% emerged for this information. Participants belonged to different medical specialisations (response rate of 25% among them: 11.5% were anaesthetists; 8.9% were cardiologists; 14% were surgeons, general or specific; 6.4% were haematologists; 5% were emergency room physicians; 8.9% were internal medicine physicians; 6.4% were transfusion medicine physicians; 5% were nephrologists; 8.9% were orthopaedists; 2.5% were psychiatrists; and 7.6% were radiologists). More than 80% stated that they had a COVID-19 department within their hospital and had contact with patients with/also diagnosed with coronavirus. Physicians were mostly employed in large hospitals (61.5%).

Physicians ranged in age from 25 to 70 years (M = 46.7, SD = 12.07), and 56.5% of the sample was male. Of them, 68% were First Level Medical Managers. They had 16 years’ service (M = 16.4, SD = 12.13), with a mean of 8.89 years of work in the same hospital (SD = 9.53) and a mean of 8.88 years in the same hospital ward (SD = 9.19). Physicians had an average of 40 working hours per week (M = 40.5, SD = 9.18; range 9–66).

### 2.3. Participants in Patients’ Study

A total of 152 patients were invited, and 111 of them took part in the research (all from Campania, southern Italy). The inclusion criteria concerned, in addition to the age of majority, having had a medical examination in a public outpatient clinic or department from no more than one month before the date of participation in the survey. The information was checked by means of two initial questions relating to these issues. Participants had attended a medical consultation in a healthcare structure within the last month. They ranged in age from 21 to 89 years (M = 52.6 years; SD = 14.88), and were mostly women (75.7%). They mostly (75%) had a university degree, 59% were married or cohabiting, and 65.4% were employed, of whom 54.2% had an indefinite term contract.

## 3. Results

### 3.1. Voices of Patients (P = Patient)

Some significant strings from the *txt* corpus clarified the patients’ perspectives.

On one side, patients highlighted needs and preferences related to service delivery in terms of efficiency and functional health structures. These aspects also pointed to the responsibility of public health managers:

*The value is less waiting time and more patient care*

(P5);

*The value is efficient public health*

(P92);

*The value is less bureaucracy, speed of care services, accuracy, and ease*

(P77);

*The value is having adequate structures*

(P56)

At the same time, the structural aspects’ “slides” immediately intersected with the relational one regarding support for inhabiting the healthcare facility; patients need to be adequately informed when they or their loved ones are in a hospital, either for a clinical examination or a longer hospital stay. Thus, these aspects refer to the functioning of the healthcare facility, but inevitably pass through the relationship with the organisation, and with its culture. In these moments, the healthcare actors who provide guidance and support to patients represent the organisation, and it is to this organisation that patients turn in order to understand how to navigate their care pathway:

*The value is a clear relationship between health workers and the patient: hospital admission, time of clinical exams, when how, taken to a different ward when his loved ones can visit him*

(P55).

The caring process described above also extends outside the hospital. A person undergoing a course of treatment does not stop being a patient when he crosses the hospital threshold on his way out. At the same time, he is an inhabitant of the neighbourhood, a citizen, a member of his household. Once again, therefore, the need to function is intertwined with patients’ psychosocial needs. They need family doctors, referral points, adequate home care, to be informed about risks through appropriate preventive healthcare, and to be assured with a guarantee that the care they need will be provided:

*The value is local and primary care medicine*

(P31);

*The value is family physicians and public structures *(P34);* better home care*

(P49);

*The value is guaranteed care *(P10);* prevention*

(P11).

Patients strongly underlined the importance of clear and timely information, which is inescapably linked to clear and adequate health communication. In the patients’ words, these aspects cannot ignore a “baggage” of social competence and training that physicians should always carry. The “core skills” described contain central elements such as cooperation. This invokes the reception of information and creates an enabling environment by building trust, which has also emerged as a focal point. At the same time, empathy was perceived in physicians’ meetings and the mutual respect experienced.

These aspects, reflecting the broader communication concept as a healthy relationship tool, must be accompanied by physicians’ adequate training. Therefore, for patients, these are the elements that can constitute value:

*The value is clear communication and information*

(P37);

*The value is communication*

(P90);

*The value is competence, professionalism, and empathy of the operators *(P9);* competence*

(P64);

*The value is respect, trust, and cooperation*

(P22);

*The value is having trained medical staff*

(P2).

These elements have an impact on the healthcare organisation as a whole. A patient is recognised as a person and not a “customer”, and his/her wellbeing goes hand in hand with improving the health service. The ultimate goal of these processes is for patients to be placed at the centre of a healthcare system that is tailored to them and has an interest in their quality of life:

*The value is considering patients as “patients” and not as clients*

(P62);

*The value is a better organisation and quality of care*

(P101);

*The value is the patient at the centre of the healthcare system*

(P79).

Last but not least, “respect” introduces us to a mutual relationship. Patients underline the need to perceive respect from a physician and to give respect. This aspect was expressed by physicians, too (see next paragraph):

*The value is respect for patients and health workers*

(P33).

### 3.2. Voices of Physicians (D = Physician)

Thus, respect emerged as mutual, is part of the set of relational skills, and is accompanied by the shared treatment process:

*The value is respect for patients*

(D33);

*The value is respect for physicians*

(D42).

Also, for physicians, competence emerged as central:

*The value is competence*

(D32).

Moreover, this was accompanied by specific elements that characterise professional actions, some of which align with the needs expressed by patients, such as the preparation of physicians, the building of trust, and empathy.

*The value is care, training, experience, and trust*

(D7);

*The value is empathy, collaboration, and communication*

(D54);

*The value is professionalism, competence, and empathy*

(D24).

Collaboration was also confirmed as a key aspect for physicians, together with the importance of information.

*The value is collaboration and information*

(D63).

As understood by physicians, a distinctive aspect of competence was a sense of responsibility. Physicians consciously perceive a large part of liability in the treatment process, an aspect that does not emerge from patients:

*The value is competence and sense of responsibility*

(D8);

*The value is a responsibility*

(D57).

Finally, even physicians have recognised healthcare organisations’ roles and efficient functioning, underlining the importance of services for users. Furthermore, as professionals, they emphasised the necessity of economic investment in medical research as an aspect that contributes to health value:

*Value is organisation, efficiency, users’ service*

(D41);

*Value is investment in research*

(D65).

### 3.3. Word Association Analyses from Patients and Physicians

As described (see Section 3.1 and Section 3.2), more aspects were common between patients and physicians.

The lemma competence (13), in line with the literature [9,10,11,12], identified competence as closely related to the values of patients and physicians. Some central competencies for patients and physicians emerged: communication (4); cooperation (4); empathy (4); and professionalism (11). Several lemmas identified the elements that, for patients, attribute value to the healthcare system (5): reduced waiting times (9); more time devoted to medical consultation (11); access to facilities (9); ease of reservation (4); a healthcare environment characterised by hospitality (4); assistance (7); and adequate care (5). All this contributes to the efficiency (6) of a service, and thus to its value. The terms *Physicians* (11) and *Patients* (11) suggested that patients and carers are key figures in the practices and activities of sharing and creating value in healthcare, respecting (9) each other. Directly linked to the context was the lemma research (5), which, especially for physicians, represented value, and the lemmas related to investment in public health (7), (4) the lack of local services (9), and proximity (4) which were needs felt by patients as well as by those who manage a large workload daily. Finally, access to information (4) is needed to control one’s own health.

The lemma organisation (11) was presented in its structural aspects with patients and in its cultural aspects with physicians (see Figure 1 and Table 1).

## 4. Discussion

The shift to outcome-based and performance-rewarded healthcare has mainly focused on physical health. However, new health paradigms, the recent pandemic, and the resulting mental health crisis call for new solutions, and value-based healthcare could be the answer, if also considered from a psychosocial perspective. According to the literature [7,8], research focused on non-economic aspects of value in healthcare is lacking.

Our study aimed to explore the psychosocial aspects of value in healthcare from physicians’ and patients’ points of view.

From the results, although healthcare physicians are moving toward patient-centred medicine [24], recognising the important role of competence and relationships between healthcare actors [25,26], from the analysis of conversations, an important difference with patients emerged: physicians focused on aspects of functioning and some relationship management skills (e.g., empathy and communication), while patients focused on the relationship between themselves and their physicians.

However, we identified the need, expressed by physicians, to improve their training and competence in their professional practice. In terms of spillover, this would positively affect the healthcare organisation and, thus, the organisational culture [27].

At the same time, they considered it essential for a service to be efficient both for themselves and their patients in terms of space, waiting time, and accessibility. Organisations are characterised by cooperation and competence for oneself and others, especially among colleagues. Perceiving oneself as competent can be supportive for health professionals. In addition, although not the specific subject of the survey, these elements may have been reinforced by handling the health emergency, which made these aspects even more relevant [3].

Patients expressed the need for some functional aspects that represent value in healthcare: shorter waiting times to make visits and reservations, less bureaucracy, speedy responses and accurate services, and guaranteed care were relevant elements to people who were treated in healthcare organisations. In relation to this it would be opportune to share a reflection, beyond the results, to link our evidence to a specific context and contingency, as is usual in organisational psychology perspectives. In different health systems, these aspects could appear “ordinary”. We must consider the contingency of the healthcare system in southern Italy to understand the importance of these statements; in the Campania region, the basic levels of healthcare are among the worst in Italy. Generally, no Southern region appears in the top ten, and Campania is the third-last (58.2%) compared to the rest of the Italian regions, which range from the most virtuous regions, such as Emilia-Romagna, Tuscany, Veneto, and Piedmont (range 91–87%) to those at lower levels than Campania, such as the Autonomous Province of Bolzano and Sardinia (range 55–57%). In the decade 2010–2019, the Campania region had the lowest percentage of guaranteed care for its citizens, as emerged from the data of the Ministry of Health, elaborated by the *Gimbe Foundation* [28]. Describing the health context of our research can further clarify our participants’ answers.

Relating to health organisational culture, values’ services are represented by territorial and primary care medicine, family physicians, and better treatment at home. Furthermore, accessible public services, adequate structures, and all the aspects put the patient at the centre of the health system. Patients consider the value of health to encompass competent physicians in terms of training, and soft skills such as professionalism, empathy, cooperation, communication, trust, and receiving clear information. A clear relationship with physicians regarding hospital admissions as patients or caregivers is also important, in order to understand the time and space of hospitalisation, clinical examinations, visits in the ward, and supporting figures. Moreover, considering patients as “patients” and not as clients, respecting themselves and health workers, is also valued. From this perspective, the central role of the health relationship emerged [29]. Our results align with literature that highlights how the needs of physicians and patients “talk to each other”, like communicating vessels; physicians who try to involve patients via the spillover effect [30] can satisfy two types of needs in patients. These are informational needs (e.g., receiving clear information and clear communication) and emotional needs (e.g., empathy).

These elements also emerged from our study as representing the value in healthcare. Specifically, communication behaviours can improve the quality of the physician–patient relationship, e.g., clear information and showing empathy, and implementing these behaviours can increase patient engagement [24] (p. 504). The positive effects of these processes also concern the organisation regarding recurrence rates, a reduction in the number of diagnostic tests performed, and access to health facilities. This is relevant, considering that a healthcare organisation evaluates value as achieving the best possible results with the lowest sustainable expenditure (Value-Based Health Care) [18].

Moreover, recent studies [24] assert that healthcare costs can be reduced by promoting a patient-centred approach and communication. Furthermore, specifically for outcomes of value in the healthcare process, the literature has highlighted for some time that patients evaluate their experience with the healthcare organisation mainly through the physicians’ communication skills [31] as perceived by patients. Part of the literature [32,33] highlights there is still a “delay” in understanding the importance of the relational function performed by communication in the health sector, representing an important training gap with which organisations must cope.

Our results went in the direction of having identified a number of relational and social characteristics as pillars of value for both physicians and patients. A meeting point can also be a starting point for a new cultural paradigm. The research showed the potential psychosocial factors related to healthcare value that could help reduce costs and improve healthcare relationships. At the same time, these findings were a first step in filling the gap in the literature regarding value from a non-economic perspective, and its undefined and unmeasured nature.

Future studies, expanding the number of participants and without limitations due to the pandemic emergency, could focus on the specific role of soft skills. The survey was carried out remotely, when all professional and social lives had been shifted online, which may have created crowding that influenced our results. Participants would have preferred to talk to a researcher face-to-face. We are aware that the approach adopted during the COVID-19 pandemic prevented us from formulating more specific questions, e.g., constructing a semi-structured interview that would investigate particular aspects of our interest and components of qualitative research, which would involve asking further questions to expand or clarify an answer. The lack of opportunity to develop an interaction influenced the responses.

On the other hand, contacting the physicians involved in the emergency was difficult, as witnessed by the few practitioners involved. It is no coincidence that the participants were all from Campania, where it was easier to move around to gather responses. Furthermore, only the target group of physicians was considered for two main reasons: (1) other professionals, such as nurses, may have professional characteristics that require a dedicated study; (2) during the COVID-19 pandemic, we could not reach the target group of nurses in public hospitals. Further investigations are ongoing to expand the number of participants and consider other healthcare professionals. Lastly, due to the advent of the pandemic, it was obligatory to use a self-report tool to collect qualitative data, in addition to the change of method in the run-up.

Despite these limitations, from an organisational perspective, innovative actions aimed at improving competence and soft skills. These aspects could influence the value perceived by physicians and patients, creating value in healthcare. Developing a culture oriented towards sharing and involving the patient in healthcare and academic contexts should be considered, and a privileged tool of this process is communication, since it is preparatory to developing solid therapeutic relationships at the service of the organisational process [34].

## 5. Conclusions

Over the past decade, discourse on value-based health care (VBHC) has been hugely popular in both public and private health care.

At the system level, healthcare systems strive to use the wellbeing of patients to assess the performance of care for full courses of treatment for a condition, but how much does this really matter to them? At the patient level, professionals aim to organise integrated care around a health condition, often making personal values prescriptive to guide treatment decisions. On this basis, our study aimed to highlight the factors that could determine the value increase in healthcare from a non-economic perspective, questioning physicians and patients. Care management as a value is more focused on achieving set goals, with maximum efficiency and accountability for outcomes. But the role of the *doctor–patient* relationship is central to value creation, whether the focus is on rules and procedures or outcomes and results, because in building a culture oriented towards care and its value, managers and citizens should play a mutually beneficial role.

From an organisational point of view, value in healthcare provides opportunities and enables the delivery of necessary social information in all areas of the world. Governments worldwide should facilitate citizens’ access, improve treatments, and provide basic territorial services. Governments in the post pandemic era should promote public awareness of policies and programs, approaches, and strategies regarding the value of healthcare [2].

Reference [35] highlights that promoting the effective development of healthcare’s value is necessary to guarantee a long-term economic commitment, and is not only linked to the single emergency of information and communication towards citizens. This process requires knowledge of the general context in which healthcare facilities operate to set realistic and achievable goals.

Serious and differing access divides exist worldwide in healthcare values. In many geographical areas of the world, the value of healthcare is guaranteed by specific laws, but these do not always ensure healthcare as a value for all citizens. Economic problems often highlight difficulties in accessing how the service is provided. In this scenario, is necessary to consider the relationships created between the medical staff, medical and nursing staff, and between these professional figures and the patients (who are citizens before they are patients) [35]. The value of healthcare should address the issues of access divide and promote opportunities for the economic and health engagement of citizens [3]. Some needs that emerged from our study (e.g., “local and primary care medicine”; “family physicians and public structures”) were in line with the recent developments related to value in healthcare [35] (p. 684) that support the ways in which the partnership creates value to share information with patients, supporting physicians in isolated places as they provide patients with care close to home. From this perspective, partnership is considered to be “natural” because of “the shared goals of creating high value and achieving better health outcomes for patients, family members, and healthcare workers” (p. 684).

As has emerged from this exploratory study, it would be interesting to invest resources in strengthening variables such as cooperation, communication with the patient to guide the care pathway, professionalism, and training as organisational support. These and other aspects can contribute to creating value in healthcare. Promoting value in healthcare means developing economic and relational investments, focusing on and enhancing the interaction and exchanges between healthcare organisational actors. Value in health is a multi-professional activity and thus is central to implementing aspects of professionalism that, as emerged from our results, are necessary to a culture of stewardship: focusing only on cost-cutting is insufficient [2].

Healthcare systems “do not have the workforce or the buildings to continue to operate traditional models of care with the growing caseloads of chronic disease management” [2] (p. 213). As is well known, without interaction, there is no culture and, therefore, no value-creating processes as a specific dimension of organisational culture [36].

Exploring psychosocial value in healthcare can have several practical implications to enhance patients’ overall wellbeing and outcomes. These aspects can create an environment that supports and empowers healthcare organisations and can be integrated into healthcare practices to support the processes of psychosocial value.

Patient-centred care: First of all, from a patient-centred perspective [24], the need to strengthen physicians’ skills in focusing on the patient relationship and an organisational culture that promotes patient-centredness and engagement emerged.

To achieve an effective organisational model that is psychosocial and patient-centred, the physician should ‘embrace’ patient-centredness as a value. When this does not happen, it creates an obstacle to implementing a psychosocial values-based healthcare culture. Moreover, reference [15] highlighted that physicians’ orientation toward psychosocial needs and the engagement of patients had a protective role against negative health outcomes, and that low levels of physicians’ skills could inhibit the health engagement process in patients, with a negative influence on the building of health organisational culture [3].

Emphasising psychosocial value in healthcare involves recognising and addressing the holistic needs of patients, including their psychological, emotional, and social well-being [25]. By adopting a patient-centred approach, physicians (and also the other specific healthcare workers in related roles) can actively involve patients in their care, consider their preferences, values, and goals, provide support beyond medical treatment [3], and incorporate these factors into the care plan.

Collaborative care: Collaboration between healthcare providers from different disciplines, such as physicians, nurses, psychologists, social workers, and occupational therapists, can promote a comprehensive and integrated approach to patient care. By working together, these professionals can address a patient’s physical and psychosocial health, leading to better overall outcomes [15].

Communication and empathy: Effective communication between physicians and patients is crucial for understanding and addressing psychosocial needs. Encouraging physicians, and other healthcare professionals, to develop strong communication skills, active listening, and empathy can help build trust, establish therapeutic relationships, and provide emotional support to patients [14].

Health literacy and education: Providing patients with education and resources to manage their health conditions can empower them to participate in their care actively.

Educating patients about the psychosocial aspects of their condition, such as stress management techniques, coping strategies, and social support networks, can help them navigate the challenges they may face and improve their overall wellbeing [27].

Outcome measurement and research (Related to this point, as aforementioned (see the section “*Aim of the Study*”), the present study is a part of a macro-project that aimed to develop a measurement addressed to physicians, and also ongoing to patients, to detect psychosocial aspects of value in care. The first tool’s validation has been closed of February 2023 and was related to physicians, and we are assessing the scale validation paper) [7,8]: Beyond the quality improvement initiatives to provide valuable insights into the effectiveness of interventions and treatments, healthcare organisations can identify areas for improvement and develop evidence-based practices that prioritise patients and, as *spillover* [30] effects, physicians’ psychosocial wellbeing, by measuring and monitoring psychosocial factors.

In conclusion, integrating the psychosocial dimensions of value into healthcare practices can improve patient and healthcare worker satisfaction, treatment plan adherence, and overall health outcomes. By recognising the interconnectedness of physical and psychosocial health, healthcare systems can provide a more holistic approach to managing care and to organisational actors that contribute to building healthcare services.

## Figures and Tables

**Table 1:** Co-occurrence analyses and cosine index (T-Lab 21).

Occurrence of EC ^1^	Frequency (4)	Cosine
Competence	13	0.283
Physicians	11	0.261
Patients	11	0.261
Organisation	11	0.261
Professionalism	11	0.261
Medical consultation	11	0.236
Waiting	9	0.236
Respect	9	0.210
Structures	9	0.236
Timing	9	0.236
Local	9	0.236
Assistance	7	0.208
Healthcare	7	0.208
Efficiency	6	0.192
Service	6	0.192
Humanity	6	0.192
Care	5	0.176
Research	5	0.176
System	5	0.176
Hospitality	4	0.157
Cooperation	4	0.157
Communication	4	0.157
Empathy	4	0.157
Information	4	0.157
Reservation	4	0.157
Proximity	4	0.157
Public	4	0.157

^1^ Elementary contexts.

**Table 2:** Association indexes between lemmas.

LEMMA	ASS (A)	ASS (B) *	COCC	χ^2^	(p)
Organisation	11	1	1	0.01533618	0.901
Professionalism	11	3	3	4.337246	0.037
Waiting	9	1	1	0.1019813	0.749
Timing	9	1	1	0.1019813	0.749
Respect	8	1	1	0.1871049	0.665
Assistance	7	1	1	0.3135378	0.576
Humanity	6	4	4	16.36234	0
Care	5	1	1	0.7710547	0.38
Service	5	1	1	0.7710547	0.38
System	5	1	1	0.7710547	0.38
Hospitality	4	1	1	1.182895	0.277
Communication	4	1	1	1.182895	0.277
Empathy	4	2	2	6.062888	0.014
Information	4	1	1	1.182895	0.277
Reservation	4	1	1	1.182895	0.277

* ASS (B) (lemma) is associated with ASS (A) (lemma); Chi Square (χ^2^) value concerning the co-occurrence (COCC) significance; p = probability associated with Chi Square value (def = 1).

## Data Availability

The data presented in this study are available on request from the corresponding author. The data are not publicly available due to the study is a part of a macro-project that is still ongoing.
